# *Pasteurella multocida* urinary tract infection in a patient with cervical cancer

**DOI:** 10.1099/jmmcr.0.005082

**Published:** 2017-01-31

**Authors:** Manmeet B. Singh, Amanda T. Harrington

**Affiliations:** ^1^​ Department of Pathology, University of Illinois at Chicago, Chicago, IL, USA; ^2^​ Department of Pathology, Loyola University Medical Center, Maywood, IL, USA

**Keywords:** urinary tract infection, *Pasteurella multocida*, cervical cancer

## Abstract

**Introduction.** Infections caused by *Pasteurella* species are commonly associated with contact with dogs and cats, typically involving bites and scratches, but casual contact with household pets can also be a risk factor. Urinary tract infection (UTI) caused by *Pasteurella* species is rare and a significant majority of cases have some known risk factor associated with an underlying chronic illness or structural and/or functional urological abnormality.

**Case presentation.** Here, we present a case of a UTI due to *Pasteurella multocida* in a patient with squamous cell carcinoma of the cervix who also had a household cat.

**Conclusion.** Providers and laboratorians should be aware of risk factors associated with UTIs caused by *Pasteurella* species.

## Abbreviation

UTI, urinary tract infection.

## Introduction

*Pasteurella* species are among the most prevalent commensal organisms found worldwide in domestic animals. Belonging to the family *Pasteurellaceae*, which includes a diverse group of small Gram-negative bacilli primarily isolated from animals, *Pasteurella* species are commonly cultured from the oral cavities of cats and dogs. *Pasteurella multocida*, the most frequently isolated species of the genus, is associated with both chronic and acute infection, although overall mortality remains low. The majority of human infections are wound infections associated with cat and dog bites and scratches; however, the clinical spectrum of *P. multocida* infections may also include the following: septic arthritis and osteomyelitis, prosthetic joint infection, meningitis, respiratory tract infections in patients with underlying airway or lung compromise (e.g. chronic obstructive pulmonary disease, lung carcinoma), endocarditis, sepsis and bacteraemia, and colonization of the lower female genital tract associated with serious gynaecological and perinatal infections (e.g. meningitis in the newborn) [1–3]. Nearly all reported cases of infection have evidence of prior animal exposure or contact, and even benign contact with animals (kissing or licking of abrasions or mucosal surfaces) can result in infection. Despite its wide clinical spectrum of infection, *P. multocida* associated with urinary tract infection (UTI) is considered rare [4, 5]. Only 14 prior cases of *P. multocida* UTI have been reported. A significant majority of these cases had some known risk factor associated with an underlying chronic illness or structural and/or functional urological abnormality [4].

## Case report

A 48-year-old Caucasian female with cervical cancer presented as an outpatient to her gynaecological oncologist at the University of Illinois Hospital and Health Sciences System (Chicago, IL, USA) 1 week after her first dose of cisplatin for follow up with her chemotherapy–radiation treatment plan. She also complained of brownish vaginal discharge, dysuria, increased urinary frequency and occasional hot flashes; however, she denied any nausea, vomiting, diarrhoea or flank pain. She had recently been diagnosed (2 months prior to presentation) with moderately differentiated squamous cell carcinoma of the cervix after biopsy of a 7 cm cervical mass involving 75 % of the cervix. Physical examination and imaging studies demonstrated that the cervical mass did not involve the vagina and there was no evidence of metastatic disease. She had a 7 pack-year history of cigarette use, worked as a server in a restaurant and had cats at home, but denied any history of recent cat bites or scratches.

On physical examination, she was afebrile with a mildly elevated blood pressure of 147/76 mmHg (19.6/10.1 kPa), but the remainder of her vital signs were within normal limits and her physical exam was unremarkable. Urinalysis showed yellow hazy urine, 70 leukocytes per high-power field, moderate leukocyte esterase, negative nitrites, rare bacteria, a specific gravity of 1.010 and a pH of 8.0. The complete blood count revealed a white blood cell count of 7.5×10^3^ cells mm^−3^, haemoglobin of 10.3 g dl^−1^ and a platelet count of 253×10^3^ platelets mm^−3^. Bacterial culture of a voided urine specimen resulted in the isolation of >100 000 c.f.u. *P. multocida* ml^−1^ as the predominant organism, which was susceptible to ampicillin, levofloxacin, tetracycline, trimethoprim/sulfamethoxazole and ceftriaxone. The patient was treated with an initial 7 day course of trimethoprim/sulfamethoxazole (800/160 mg).

In her follow-up clinic visits within 1 week of starting antibiotic therapy, the patient continued to complain of mild dysuria, relieved with pyridium, and her antibiotic course was extended for an additional 7 days. Four subsequent urine cultures had no growth, two of which were done within 2 weeks after starting antibiotic therapy and the other two cultures were done 2–4 months after treatment, as the patient continued to complain of UTI-type symptoms; however, the symptoms were attributed to radiation cystitis. Follow up imaging studies 5 months after starting chemotherapy and radiation therapy showed no evidence of active malignancy.

## Discussion

*P. multocida* is readily recovered by standard media in the clinical microbiology laboratory, growing well on 5 % sheep’s blood and chocolate agars, but not MacConkey agar [1]. In our specific case, the organism was only recovered from blood agar, as chocolate agar was not routinely used in the urine culture protocol. These facultative small Gram-negative bacilli are typically oxidase positive, catalase positive and indole positive. The organism in our case was identified as *P. multocida* using the VITEKMS MALDI-TOF(matrix-associated laser desorption ionization-time of flight) mass spectrometry system (bioMérieux) and confirmed with 100 % match to type strains by 16S rRNA gene sequencing using methods previously described [6].

Although the aetiology of *P. multocida* UTI is not clear, patients with chronic illnesses and structural or functional genitourinary tract abnormalities appear to be at risk for infection [4, 5, 7–10]. Of the 14 reported cases of human *P. multocida* UTI, 8 cases have occurred in females, 4 of whom had an associated genitourinary malignancy (3 with cervical carcinoma and 1 with bladder carcinoma). Six cases were in males, one of whom had an associated malignancy (prostatic adenocarcinoma) [4]. Of the nine cases not associated with malignancy, seven patients were reported to have an underlying chronic illness associated with the genitourinary tract (e.g. chronic cystitis, paraplegia with chronic pyelonephritis, insulin-dependent diabetes with an indwelling Foley catheter, chronic renal failure from obstructive uropathy) [4, 7–9]. Only two cases were not associated with an underlying medical illness. One patient presented with pneumonia and no other comorbidities [8], and the other reported a 20 pack-year history of smoking and a remote history of pyelonephritis [10].

As with the majority of *Pasteurella* infections, there appears to be a strong association between infection and animal contact in UTIs. Dixon and Keresteci reported the first case of *P. multocida* UTI in a human in 1967, involving a 62-year-old female with uterine cervical carcinoma status post-radiation therapy with non-functioning kidneys and a pet cat at home [11]. The most recent case of *P. multocida* UTI was reported by Cortez *et al*. in 2007 [4], and involved a 13-year-old boy with end-stage renal disease receiving peritoneal dialysis, who had a pet cat at home. In neither case was any history of traumatic animal exposure (e.g. bites or scratches) reported [8]. In the case described by Mann and Quenzer, the patient, who presented with pyelonephritis, had a dog at home, which apparently frequented her bathroom and bedroom. The authors noted that the dog’s oral cavity had been cultured and a heavy growth of *P. multocida* was recovered [10]. Notable exceptions are those presented by Komorowski and Farmer in 1974. Although no recent animal contacts were reported in these three cases, the patients did have other reported risk factors for infection (e.g. prostatic adenocarcinoma status post-transurethral resection of prostate, insulin-dependent diabetes with an indwelling Foley catheter and insulin-dependent diabetes status post-pyloroplasty/vagotomy for recurrent peptic ulcer) [9]. Also, for the cases reported by Hubbert and Rosen in 1970 there was no specific comment on whether these patients had recent animal exposure or not, so the extent of the association is unknown [8].

Our patient had both a risk factor for infection involving a structural genitourinary tract abnormality due to her uterine cervical carcinoma and also a pet cat at home. She denied any history of recent cat bites or scratches, which is consistent with reports that *P. multocida* is the species most-often associated with infections that are not a result of a bite wounds [1]. This case also highlights the potential of frequent casual contact with animals as the source of *P. multocida* UTI. Of the six UTI cases where animal exposure was noted, each case involved casual animal contact as opposed to traumatic animal exposure [4, 5, 7, 10–12]. Liu *et al*. reported a case of *P. multocida* UTI in a 56-year-old female with uterine cervical cancer who had exposure to her pet cat along with a visiting cat [5]. The patient denied any traumatic animal exposure, and molecular methods determined that the isolate from the patient’s urine matched the isolate recovered from the oral cavity of the patient’s cat, but differed from the isolate of *P. multocida* recovered from the visiting cat [5]. These cases provide support for zoonotic transmission of *P. multocida* through frequent and repeated casual animal contact from pets in the home as a possible risk factor for sub-clinical carriage and/or infection.

*P. multocida* UTI is rare in humans. Infection is associated with an underlying chronic illness or structural and/or functional genitourinary abnormality in patients who often have pet cats or dogs at home, likely mediated through frequent casual contact, highlighting the importance of a pet-related history even without traumatic animal exposure. Providers should continue to emphasize good hand hygiene for pet owners, especially those with potential risk factors. In addition to recovery of *P. multocida* from typical wound-related specimens, laboratory staff should be aware this organism can be recovered from a variety of specimens, including urine specimens.
